# Enhanced mosquitocidal efficacy of pyrethroid insecticides by nanometric emulsion preparation towards *Culex pipiens* larvae with biochemical and molecular docking studies

**DOI:** 10.1186/s42506-021-00082-1

**Published:** 2021-07-15

**Authors:** Nehad E. M. Taktak, Mohamed E. I. Badawy, Osama M. Awad, Nadia E. Abou El-Ela, Salwa M. Abdallah

**Affiliations:** 1 Department of Tropical Health, High Institute of Public Health, Alexandria University, 165 El-Horreya Ave., 21561-El-Hadara, Alexandria, Egypt; 2 Department of Pesticide Chemistry and Technology, Laboratory of Pesticide Residues Analysis, Faculty of Agriculture, Alexandria University, 21545-El-Shatby, Alexandria, Egypt; 3 Mammalian and Aquatic Toxicology Department, Central Agricultural Pesticides Laboratory (CAPL), Agricultural Research Center (ARC), Dokki, 12618 Egypt

**Keywords:** *Culex pipiens*, Pyrethroids, Nanoemulsion, Insecticidal activity, Biochemical studies, Molecular Docking, Ecotoxicity

## Abstract

**Background:**

The growing threat of vector-borne diseases and environmental pollution with conventional pesticides has led to the search for nanotechnology applications to prepare alternative products.

**Methods:**

In the current study, four pyrethroid insecticides include alpha-cypermethrin, deltamethrin, lambda-cyhalothrin, and permethrin were incorporated into stable nanoemulsions. The optimization of nanoemulsions is designed based on the active ingredient, solvent, surfactant, sonication time, sonication cycle, and sonication energy by factorial analysis. The nanoscale emulsions’ droplet size and morphology were measured by dynamic light scattering (DLS) and transmission electron microscopy (TEM), respectively. The toxicity of nanoemulsions against *Culex pipiens* larvae was evaluated and compared with the technical and commercial formulations. The in vitro assay of adenosine triphosphatase (ATPase), carboxylesterase (CaE), and glutathione-S-transferase (GST) were also investigated. Furthermore, molecular docking was examined to assess the binding interactions between the tested pyrethroids and the target enzymes. Also, an ecotoxicological assessment of potential effects of the tested products on the freshwater alga *Raphidocelis subcapitata* was determined according to OECD and EPA methods. The emulsifible concentration (EC_50_) and NOEC (no observed effect concentration) values were estimated for each insecticide and graded according to the GHS to determine the risk profile in aquatic life.

**Results:**

The mean droplet diameter and zeta potential of the prepared pyrethroid nanoemulsions were found to be in the range of 72.00–172.00 nm and − 0.539 to − 15.40 mV, respectively. All insecticides’ nanoemulsions showed significantly high toxicity (1.5–2-fold) against *C. pipiens* larvae compared to the technical and EC. The biochemical activity data proved that all products significantly inhibited ATPase. However, GST and CaE were significantly activated. Docking results proved that the pyrethroids exhibited a higher binding affinity with CaE and GST than ATPase. The docking scores ranged from − 4.33 to − 10.01 kcal/mol. Further, the biosafety studies of the nanopesticides in comparison with the active ingredient and commercial EC were carried out against the freshwater alga *R. subcapitata* and the mosquitocidal concentration of nanopesticides was found to be non-toxic.

**Conclusion:**

The mosquitocidal efficacy of nano-pyrethroids formulated in a greener approach could become an alternative to using conventional pesticide application in an environmentally friendly manner.

**Supplementary Information:**

The online version contains supplementary material available at 10.1186/s42506-021-00082-1.

## Introduction

*Culex pipiens* is one of the many members of the disease-carrying mosquito family. Specifically, *C. pipiens* is a well-known carrier of the West Nile virus, Saint Louis encephalitis viruses, canine Dirofilaria worms, avian malaria, and filarial worms. Since the early twentieth century, campaigns have been organized in many countries to control this pest species [1]. For *C. pipiens* mosquitoes’ chemical control, pyrethroid insecticides have been extensively used worldwide [2]. Their use increased to represent from 18% in 2002 to 30% in 2017 of the total global pesticide market [3]. These pesticides are synthetically modified analogs of the essential natural pyrethrins found in flowers of the *Chrysanthemums* genus. In general, many new pyrethroids are synthesized and added to the market to meet the enhanced global demand for food, vector-borne diseases, and pest species resistant to other pesticides [4]. Pyrethroids are an essential way to combat malaria and other mosquito-borne diseases despite the risk of pyrethroids resistance in vector populations [5]. Pyrethroids are also common ingredients of household insecticides. The home environment’s unregulated use increases the risk of exposure and adverse effects in the general population [6]. Synthetic alpha-cyano pyrethroids such as alpha-cypermethrin, deltamethrin, and lambda-cyhalothrin are potent environmentally compatible insecticides and have a wide margin of safety for mammals for preferential application in agricultural, veterinary, and public health programs [7].

Pyrethroid products have been traded in some formulations, the most popular of which are emulsifiable concentrate (EC), aerosol dispenser, wettable powder, dust powder, and water dispersion granules [8]. The EC of pyrethroids is usually two to nine times more toxic than the technical grade, likely due to synergistic reactions. It is one of the most widely used delivery systems for hydrophobic pesticides, accounting for 40–50% of total formulations. However, about 300,000 tons per year of organic solvents are used to prepare the EC formulations [9]. Besides, other common solvents and co-solvents can also be used. These solvents have flammable, explosive, and toxic properties that make them harmful to humans and crops and produce poisonous residues in the environment [10]. In practice, some of the problems associated with using conventional emulsifiers as delivery systems for hydrophobic pesticides relate to their relatively large droplet size. Emulsions are dynamically unstable systems that tend to collapse through gravitational separation, droplet aggregation, and Ostwald ripening. All these factors can negatively affect the end product’s efficiency and shelf life, reducing its pest control ability. Many of these problems can be overcome with the use of nanoemulsions, which are an effective way to use pesticides efficiently, economically, and safely [11]. Nanoemulsions consist of emulsifier-coated fine oil droplets dispersed in water, having droplets covering the size range of 20–500 nm [9]. They are also referred to as mini-emulsions, ultrafine emulsions, submicron emulsions, and others. Due to their particular size, nanoemulsions are transparent or translucent to the naked eye and are stable against sedimentation or creaming. The smaller size of the droplets increases their stability to the gravitational separation and accumulation of droplets. It increases their deposition, diffusion, and permeability to plant leaves and insect body surfaces [12]. The composition, characteristics, mechanism of formation, and stability of pesticide nanoemulsions have essential theoretical and practical significances on the promotion and application of pesticides compared to conventionally applied pesticides [13, 14].

The main objectives of this study were to prepare and characterize O/W nanoemulsions of four pyrethroid insecticides (alpha-cypermethrin, deltamethrin, lambda-cyhalothrin, and permethrin). Various factors were designed and investigated to prepare the nanoemulsions using a factorial design by Minitab software. Factors include the concentration of the active ingredient, solvent, surfactant, sonication time, sonication pulses, and sonication power. The prepared nanoemulsions’ characterizations, including the droplet size distribution, polydispersity index (PDI), viscosity, pH, stability, and surface morphology by transmission electron microscopy (TEM) were investigated. The toxicity of the nanoemulsions was investigated against *Culex pipiens* larvae comparing to the active ingredient and commercial EC. Biochemical studies were also investigated in vitro on adenosine triphosphatase (ATPase), carboxylesterase (CaE), and glutathione-S-transferase (GST). We further applied the molecular docking of these insecticides in conjunction with existing experimental data and enzyme-associated tested insecticides to hypothesize how these compounds would interact with the target proteins. Further, this study demonstrated the non-toxic property of nanopesticides towards non-target species of the freshwater alga *Raphidocelis subcapitata*.

## Methods

### Tested pyrethroids

The technical grade of alpha-cypermethrin (97%), deltamethrin (98%), and lambda-cyhalothrin (95%) were obtained from Syngenta Agro. Co (6th of October, Giza, Egypt), while permethrin technical grade (96%) was obtained from Chema Industries (26 First Industrial Area, EL-Nubariya, El-Beheira Governorate, Egypt). The chemical structures and physicochemical properties of the tested pyrethroids are shown in Table S1. Formulated forms of alpha-cypermethrin (25% EC, Spar-kill®) and lambda-cyhalothrin (10% EC, Lambda®) were purchased from El-Helb Pesticides and Chemicals Co. (Dumyat Al Jadidah, Dumyat, Egypt. Deltamethrin formulation (5% EC, Nu-tox®) was obtained from Alexandria Co. for Pharmaceuticals & Chemical industries, Co**.** Permethrin formulation (25% EC, Pergon®) was obtained from MEDMAC for Manufacturing Agricultural Chemicals & Veterinary Products Ltd (Um-Mutwa’ Al-Aslameya Street, Al-Jandaweel, Amman, Jordan).

### Chemicals and reagents

Adenosine triphosphate (ATP), bovine serum albumin (BSA), 1-chloro-2,4-dinitrobenzene (CDNB), dimethylsulfoxide (DMSO), Folin-Ciocalteu phenol, L-glutathione (GSH), α-naphthyl acetate, β-nicotinamide adenine dinucleotide (β-NAD), tetrazotized O-dianisidine (fast blue B salt), trichloroacetic acid (TCA), Tris (hydroxymethyl)aminomethane), triton X-100 and tween 80 were purchased from Sigma-Aldrich Chemical Co. (St. Louis, MO, USA). Other commercially available solvents and chemicals such as ammonium molybdate, copper sulfate, EDTA (ethylenediaminetetraacetic acid), ferrous sulfate, and sodium-potassium tartrate were of analytical grade and purchased from El-Gomhouria For Trading Chemicals And Medical Appliances Co., (Adeb Ishak St, Manshia, Alexandria, Egypt) and used without further purification.

### Culture of *Culex pipiens* larvae

A susceptible strain of *C. pipiens* culture was obtained from Research Institute of Medical Entomology, Ministry of Health, Dokki, Giza, Egypt, and reared in High Institute of Public Health insectary, Alexandria University, Alexandria, Egypt [15]. The Larvae were fed on biscuits [containing wheat flour and yeast powder mixed with milk powder (10: 1: 1, w/w, respectively)] until pupation in shallow trays containing 2–3 L of de-chlorinated water. Male adults were fed on 30% sucrose solution, and females were fed on pigeon blood four times a week in adult cages. Using a live pigeon in our study was according to the Ethics Committee of High Institute of Public Health’s acceptance and proved from Alexandria University under reference number 481. The egg rafts were transferred from adult cages to white trays containing de-chlorinated water for egg hatch [16].

### Experimental design for nanoemulsions preparation

The experimental design allows studying the effect of many variables with a limited number of experiments. The design relied on deltamethrin as an insecticide chosen from among the four types of pyrethroids tested. Windows version of MINITAB 19.1 software (2019 Minitab Inc.) was used to design the experiments [17]. Statistical analysis of the results will reveal which variables have a significant influence and correlate the desired response with the variables by the polynomial equation:

Y_1_= A_0_ + A_1_X_1_ + A_2_X_2_ + A_3_X_3_ + A_n_X_n_

where Y is the dependent variable, A_0_ is a constant, and A_1_–A_n_ are the coefficients of the independent values. X_1_-X_n_ represent independent factors.

A factorial experiment consists of two or more factors from these designs, each with discrete possible values or “levels.” In this study, the factorial design methodology was used to study the effects of six independent variables: the concentration of deltamethrin (as an example of pyrethroids active ingredient), DMSO as a solvent, and tween 80 as a surfactant, in addition to applied sonication power, sonication time, and pulses of sonication on the droplet size, PDI, pH, and viscosity of prepared pyrethroid nanoemulsions. Droplet size, PDI, viscosity, and pH were determined as the dependent variables. Nine experimental trials involving six independent variables were obtained from the software. Each variable was tested at two levels, low (−) and high (+), in addition to the mean level of each variable was tested in only one experiment**.**

### Preparation of nanoemulsions

**Fig. 1:**
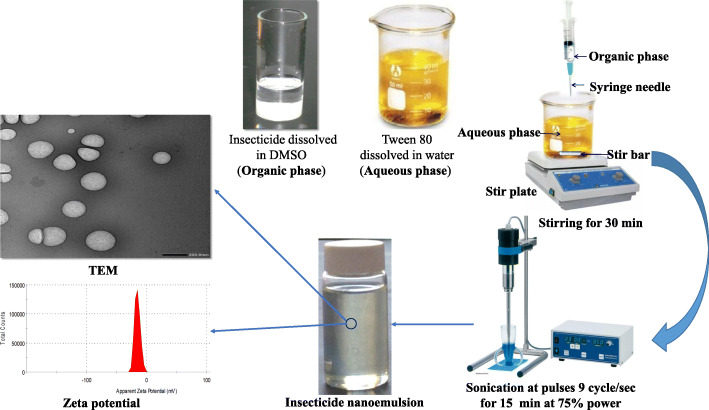
Schematic illustration of the preparation and characterization of pyrethroid nanoemulsions

To achieve the final optimized conditions, nine formulations of deltamethrin were prepared in different experimental setups, including the organic phase (active ingredient and DMSO), an aqueous phase containing Tween 80, sonication time, sonication power, and sonication pulses. Deltamethrin was used as an example of pyrethroids in optimization experiments. However, four insecticides, including alpha-cypermethrin, deltamethrin, lambda-cyhalothrin, and permethrin, were prepared by the optimum method. Briefly, 0.5% a.i (w/v) of each insecticide were dissolved in DMSO to form the organic phase. Tween 80 was dissolved in distilled water to form the polar phase. The organic phase was dropped into a polar phase to form the coarse emulsion by stirring at room temperature for 30 min at 4000 rpm. The coarse emulsion was later converted into a nanoemulsion through ultra-sonication using a high-energy ultrasonic process by the ultrasonic probe (Ultrasonic Homogenizers HD 2070 with HF generator (GM 2070), ultrasonic converter UW2070, booster horn (SH 213 G), and probe microtip MS 73, Ø 3 mm) (Fig. 1). The tip of the horn was symmetrically placed in the coarse emulsion, and the ultra-sonication process was carried out at pulses 9 cycles/sec, power 75 % for 15 min [18].

### Characterization of the nanoemulsions

#### Stability studies

The prepared nanoemulsions were subjected to stability screening tests to select the most stable formulation. These stability tests, including centrifugation assay stability at a temperature of 25 and 40 °C and heating-cooling test. Centrifugation assay in which three samples from each prepared formulation were centrifuged for 30 min at 5000 rpm and noticed phase separation, creaming, and cracking. The nanoemulsions should have enough stability without phase separation. Stable formulations were exposed to other thermodynamic stability tests [19]. About 25 mL of freshly prepared nanoemulsions were transferred to a transparent tube. The transformation from a steady state to creaming and coalescence was examined during the storage period of 3 months at 25 °C. After that, the heating-cooling test was investigated to show the effect of heating and cooling on the prepared nanoemulsions’ stability. The prepared nanoemulsions were maintained at a temperature of 4 °C and 40 °C with storage for 48 h for each temperature test. The formulations that remained stable at this temperature were subjected to further investigation.

#### Droplet size, polydispersity index, and zeta potential

The droplet size, PDI, and zeta potential of pyrethroids nanoemulsion formulations were investigated using Zetasizer Nano ZS (Malvern Instruments, UK) at room temperature. The mean particle size and PDI of nanoemulsions were measured by the dynamic light scattering (DLS) technique. Emulsion droplet size was estimated by the average of three measurements and presented as mean diameter in nm, while zeta potential was determined by the light scattering method [20]. The formulations were diluted with distilled water by 200-fold and sonicated for 5 min at pulses 9 cycles/s and 75 % power before the measurement to avoid multiple scattering effects.

#### Transmission electron microscopy

Surface morphology, topology, and droplet size of four pyrethroids nanoemulsions were characterized by TEM (JEOL JEM-1400 Plus TEM, USA, Inc.) equipped with a 20-mm aperture at 20 kV. Bright-field imaging increasing the magnification and diffraction modes was selected to reveal the nanoemulsions’ form and size. The nanoemulsion of each pyrethroid formulation (10 mL) was diluted with distilled water (1/100) and added to 200-mesh form war-coated copper TEM sample holders (EM Sciences, Hatfield, PA, Japan).

#### Viscosity and pH measurements

The dynamic (absolute) viscosity of the nanoemulsion was determined using a digital viscometer (a Rotary Myr VR 3000) with an L3 spindle at 200 rpm at 25 °C. The viscosity of the formulations was measured without further dilution. Each reading was recorded after the equilibrium of the sample for 2 min. The viscosity recording of samples was repeated three times, and the data expressed in mPa.s. In the present study, the digital pH meter (Crison pH Meter Basic 20, EU) was used to determine the prepared nanoemulsions’ pH values.

### In vitro release of pyrethroids from nanoemulsions

In vitro release experiments were carried out using the dialysis technique [21]. Two milliliters from each formulation (0.5%, v/v) was placed inside a dialysis bag (cellulose membrane, molecular weight cut-off 14,000, Sigma-Aldrich Co., St. Louis, MO), sealed, and immersed in a vessel containing 50 mL of 10 mM phosphate buffer solution (pH 7.4). The releasing system was maintained at 37 ± 1 °C under magnetic stirring (100 rpm). One milliliter from the solution was taken out of the dissolution medium at predetermined time intervals, replaced with fresh buffer solution. Pyrethroids released were determined by ultra-high-performance liquid chromatography (UHPLC, UltiMate 3000 system, Thermo Scientific, USA). The system was equipped with a DIONEX UltiMate 3000 variable wavelength ultraviolet detector (VWD). The separation was performed on analytical column ODS Hypersil C18 (250 × 4.6 mm diameter, 5-micron particle size, Thermo scientific, USA). Data were managed using a Chromeleon™ Chromatography Data System Software. The system consists of a binary gradient solvent pump to control the mobile phase’s flow rate and an autosampler for automatic injection, a vacuum degasser, and a column oven (5–80 °C). The detection of tested pyrethroids was with a flow rate of 1 mL/min, injection volume of 10 μL, and gradient solvent system, as shown in Table S2. The tested pyrethroids’ release profile was expressed as a cumulative concentration (mg/L ± SE) and plotted versus time. The experiments were carried out in triplicate for each tested compound. The analytical grade of tested pyrethroids was used for standard preparation. The calibration curve obtained from each insecticide’s analytical standard was used to determine the final concentrations released from the nanoemulsions.

### Toxicity assay against *C. pipiens* larvae

According to the World Health Organization, the larval bioassay was performed to compare the effect of nanoemulsions of selected pyrethroids with their active ingredient and commercial EC formulations on the *C. pipiens* larva recommendations [16]. Third instar larvae were used in the evaluation by a direct contact method. The three forms of tested insecticides (technical, commercial EC formulation, and nanoemulsion) were tested to obtain the LC_50_ values. Technical pyrethroids were dissolved in DMSO and mixed with Tween-80 (0.05%), while the EC and nanoemulsions were dissolved in distilled water. Different concentrations ranging from 0.5 to 500 μg/L were tested in three replicates. Twenty *C. pipiens* larvae were put into plastic cups containing 100 mL of de-chlorinated water. The larvae were treated separately with Tween 80 or DMSO, and larvae without any treatment were maintained as control. The larvae’s morbidity and mortality were verified and recorded based on the larvae’s uncoordinated movement after investigating the cervical region with a needle. Larval mortality percentages were recorded after 24 and 48 h, and the median lethal concentration (LC_50_) values were calculated from probit analysis with 95% confidence limits and other statistical parameters [22].

### Biochemical studies

#### Preparation of enzyme homogenates and total protein assay

Surviving larvae were homogenized in 10 mM NaCl (1%, w/v) Triton X-100, and 40 mM sodium phosphate buffer (pH 7.4) at 4°C to determine Adenosine triphosphatase (ATPase), carboxylesterase (CaE), and glutathione-S-transferase (GST) activities after 24 h of exposing to LC_50_ values of the tested pyrethroids. The homogenate was centrifuged at 5000 rpm for 20 min at 4°C. The supernatant was used immediately for enzymatic assay or stored at – 20 °C. Total protein was determined according to Lowry et al.’s [23] method, and the concentrations were calculated by comparing with the standard curve of BSA.

#### ATPase assay

ATPase activity was performed according to Koch’s [24] method. The reaction mixture, which contained 400 mM Na^+^, 20 mM K^+^, 5 mM Mg ^+,^ and 5 mM ATP, was prepared, and 200 μL of the crude enzyme was added to this mixture. Then, the volume was completed to 950 μL with Tris-HCl buffer (pH 7.4). After 10 min incubation at 37 °C, the reaction was stopped with 200 μL of TCA. A fresh color reagent (5 g ferrous sulfate in 10 mL ammonium molybdate solution prepared in 10 N sulfuric acid) was added to the reaction mixture. The absorbance of the developed blue color was measured at 740 nm, and the enzyme activity was calculated as OD_740_ min^−1^ mg protein^−1^.

#### CaE assay

CaE activity was determined according to Van Asperen’s [25] method, which used α-naphthyl acetate as a substrate. The assay mixture contained 50 μL of homogenate enzyme, 2.1 mL of 50 mM sodium phosphate buffer (pH 7.4), and 25 μL of 5 mM α-naphthyl acetate solution. The mixture incubation was done at 37 °C for 15 min. Finally, 25 μL of 0.3% Fast blue B salt dissolved in 3.5% SDS was added and incubated for 15 min at 37°C. The absorption was measured at 555 nm. The enzyme activity was expressed as OD_555_ min^−1^ mg protein^−1^.

#### GST assay

GST assay was performed using reduced glutathione (2.5 mM) by Saint-Deniset et al. [26]. The assay mixture contained 100 μL of 1.5 mM CDNB, 200 μL of reduced glutathione, and 1.5 ml of pH 7.4 phosphate buffer. A total of 200 μL of the enzyme was added to the above mixture, shaken gently, and incubated for 15 min at 37 °C. The absorbance was recorded at 340 nm using a UV/Visible spectrophotometer (Alpha-1502. Laxco Inc, USA). One unit of enzyme activity attributed to the quantity of conjugated enzyme with 1 mmol of GSH per min. The enzyme activity was expressed as OD_340_ min^−1^ mg protein^−1^.

### Molecular docking

The modeled protein structure, ATPase (PDB ID: 4BYG) and detoxifying enzymes CaE (PDB ID: 5W1U) and GST (PDB ID: 5FT3) in their PDB formats were downloaded from the protein data bank (PDB) (http://www.rcsb.org) and imported on to the Molecular Operating Environment (MOE) 2014.13 software (Chemical Computing Group Inc, Montreal, Quebec, Canada). The structure of each enzyme was visualized by the MOE [27]. The protein chemistry of the missing hydrogen was corrected, after which the heteroatoms and the crystallographic water molecules were removed from the protein. *Chemical s*tructures of the tested pyrethroids were drawn by ChemDraw Professional Ultra Version 15 (PerkinElmer, Informatics, Inc., USA). The structures were converted to 3D, and the energy was minimized by the MMFF94 function [28]. The triangle-matching algorithm was selected from MOE for docking the compounds into the selected enzymes’ active sites. Free energy of binding was calculated from the contributions of hydrophobic, ionic, hydrogenated, and van der Waals interactions. A ligand was considered adequate for a minimum docking score value (or interaction energy calculation) of an enzyme-ligand complex.

### Bio-efficacy experiment on the freshwater green alga

The freshwater green alga *Raphidocelis subcapitata* was obtained from the Faculty of Science; Mansoura University, Egypt. The stock culture was maintained in 250-mL borosilicate Erlenmeyer flasks containing culture medium at 24 ± 2 °C, under a continuous white fluorescent light of 3000–4000 lux, and manually shaken twice a day [29]. The axenic culture was maintained for the provision of a continuous supply of “healthy” cells for the tests in a standard algal assay medium (AAM) as described in Miller et al. [30].

An acute algal growth inhibition test was conducted using different concentrations of each insecticide in sterile AAM in a final volume of 50 mL. Tested concentrations of a pesticide were prepared from stock solutions on an arithmetic progression covering an expected range of toxicity from 0 to 90%. Stock solutions of technical insecticides were prepared in 1% DMSO and a corresponding control was included. Stock solutions of the EC and NE were prepared. Algal suspensions were exposed to different concentrations (0.0005–500 mg/L) prepared in the same medium of algae culture. All assays were conducted in triplicate. An inoculum of the exponentially-growing culture of *R. subcapitata* (harvested from 4–7 days stock culture) was prepared no more than 2–3 h before the beginning of the test. Initial cell density for the growth inhibition test was 10,000 cells/ml in both test and control flasks. Zero-time begins at inoculation of all flasks with the algal cells followed by incubation for 96 h in a temperature-controlled (25 °C) orbital shaker set at 100 rpm under continuous illumination via white fluorescent lamps. After 96 h, algal growth in terms of viable cell concentration was examined in a Neubauer hemocytometer using a phase-contrast microscope. Growth rate inhibition of the alga was used as the endpoint in this assessment. The percent inhibition values were calculated after 96 h, and the median effective concentration (EC_50_) values were calculated from the probit analysis with 95% confidence limits [22]. The no observed effect concentration (NOEC) after algal exposure to each tested insecticide was calculated by the formula: NOEC = EC/10 [31]. Furthermore, the hazard statement of each tested insecticide was estimated according to UNECE GHS (2019) [32].

### Statistical analysis

Statistical analysis was performed using the IBM SPSS software version 25.0 (SPSS, Chicago, IL, USA) [33]. Mortality percentages were calculated for each treatment and corrected using Abbott’s equation [34]. Means and standard error (SE) were obtained from three independent replications performed for each treatment. The log dose-response (LdP) lines were used in the determination of the LC_50_ values for the mosquito’s bioassay and EC_50_ values for the algal bioassay according to the probit analysis [22]. The least-square regression analysis was used to determine the 95% confidence limits. Analysis of variance (ANOVA) of the biochemical data was conducted and means property values were separated (*p* ≤ 0.05) with Student-Newman-Keuls (SNK).

## Results

### Physiochemical properties of the tested pyrethroids

The chemical structure and physicochemical properties of the tested pyrethroids are shown in Table S1. Permethrin from type I pyrethroids lacks a cyano group and three insecticides from type II pyrethroids (alpha-cypermethrin, deltamethrin, and lambda-cyhalothrin) in which an alpha-cyano group is present at the phenyl benzyl alcohol position. The tested compounds’ molecular weight was 416.3, 505.21, 449.85, and 505 g/mol for alpha-cypermethrin, deltamethrin, lambda-cyhalothrin, and permethrin, respectively. The polar surface area (PSA) of all tested pyrethroids was 59.32, except permethrin was 35.53, while the hydrophobicity factor (ALogP) of all tested compounds was around 6. There are no hydrogen bond donors (HBD) in the tested pyrethroids, while the number of hydrogen bond acceptors (HBA) ranged from 3 to 7.

### Optimization of the nanoemulsions preparation

The different experimental setup using Minitab software was used to determine the influence of six independent variables on the pyrethroid nanoemulsions’ characterization (dependent variable) (Table S3). Deltamethrin was selected as a model of the tested pyrethroids for the optimization experiments. During the nanoforming process, emulsification was achieved in the context of the droplet shearing phenomenon. The sound waves (frequency 25–75 Hz) generated by the sonotrode (a tool that creates ultrasonic vibrations) were applied to induce a mechanical vibration. Followed by acoustic cavitation, which could lead to a further collision and cause strong shock waves to shear the largest droplets to a nanometer size. The visual appearance of the nine deltamethrin nanoemulsions is shown in Figure S1. The quantitative results including the droplet size (nm), PDI, and viscosity (mPa.s) are presented in Table S4. There are significant differences in the droplet size of the nine prepared deltamethrin formulations. Formulations 1, 5, and 7 presented 172.46, 364, and 417 nm, respectively, while the other six formulations showed droplet sizes larger than 500 nm. In the PDI case, there are no significant differences between the formulations (0.516–0.964) except formulation 2 (PDI = 0.158). Nanoemulsions were exposed to extreme storage conditions to predict the samples’ ability to be physically stable for up to three months. All prepared deltamethrin formulations did not pass the centrifugation test at 5000 rpm except formulations 1 and 5, while all prepared formulations did not pass the heating-cooling test. The viscosity and pH measurements of prepared deltamethrin formulations. There is no significant difference between the viscosity values of formulations 1, 2, 5, and 6 (74.67, 80.16, 90.23, and 90.23 mPa.s, respectively). Formulations 3 and 7 have 40.32 and 37.67 mPa.s, respectively. However, formulations 4, 8, and 9 showed significant differences in their viscosity values (81.00, 160.23, and 70.67 mPa.s, respectively). The pH of the prepared formulations was in the range of 7.78-8.18.

Based on these quantitative data, the first-order polynomial equations (1-4) and their corresponding coefficients were generated for each of the response variables in the factorial design test. The models indicated the individual parameters’ behavior in others’ presence on the viscosity, particle size, PDI, and pH for deltamethrin nanoemulsions.

**Table:** 

Viscosity = − 110 + 21.09 a.i − 0.67 solvent + 5.11 surfactant + 9.86 sonication pulses + 1.91 sonication time + 0.255 sonication power s = 23.0714, r^2^= 89.49%	……….(1)
Droplet size = 20951 + 1524 a.i − 180 solvent − 514 surfactant − 417 sonication pulses − 216 sonication time + 25.5 sonication power s = 1959.47, r^2^= 87.19%	……….(2)
PDI = − 0.76–0.0336 a.i + 0.0256 solvent − 0.0010 surfactant + 0.0541 sonication pulses + 0.0244 sonication time − 0.00548 sonication power s = 0.2112, r^2^ = 82.45%	……….(3)
pH = 6.52 − 0.0187 a.i + 0.0304 solvent + 0.0155 surfactant − 0.0006 sonication pulses − 0.0048 sonication time − 0.00235 sonication power s = 0.1863, r^2^ = 62.06%	……….(4)

**Fig. 2:**
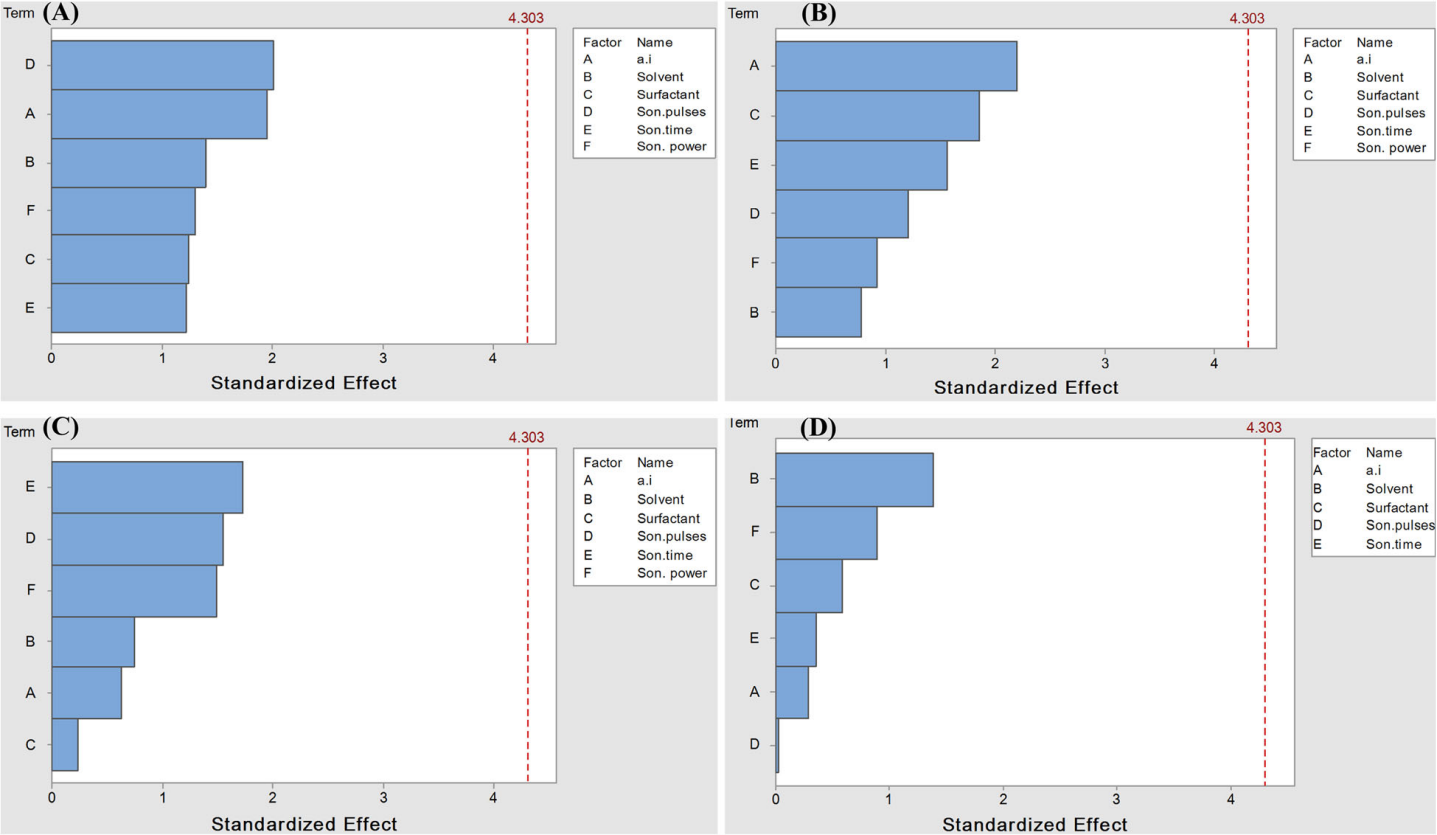
Pareto charts representing the effect of factors and process variables on viscosity (**A**), droplet size (**B**), PDI (**C**), and pH (**D**) for deltamethrin nanoemulsions at α = 0.05

Also, the influence of each factor on the response variables was shown as Pareto charts in Fig. 2. It was noted that the active ingredient, sonication pulses, and surfactant were more significant factors than the others on the nanoemulsion viscosity (Fig. 2A). In comparison, the active ingredient, surfactant, and sonication time showed the highest effect on the prepared nanoemulsions’ particle size (Fig. 2B). In the case of the PDI value, the sonication power, sonication time, and sonication pulses, respectively, had a significant effect (Fig. 2C). On the contrary, the solvent was the most significant factor in the pH value (Fig. 2D).

Among the 9 different experimental setup (Table S3), formulation 1 with 0.5 % a.i, 44% DMSO, 15% tween 80, 40.5% water, 9 cycle/s sonication pulses, and 75% sonication power for 15 min was the best. The resulted nanoemulsion 1 was in clear visual appearance with a smaller droplet size of 234 nm ± 4.13. Therefore, these parameters were selected to prepare the other pyrethroids nanoemulsions (alpha-cypermethrin, lambda-cyhalothrin, and permethrin**)**.

### Characterizations of the pyrethroid nanoemulsions

#### Droplet size and polydispersity index

The droplet size of alpha-cypermethrin, deltamethrin, lambda-cyhalothrin, and permethrin nanoemulsions were 90.26, 172.00, 168, and 72, respectively (Table 1). However, the PDI values were 0.337, 0.827, 0.448, and 0.295 for alpha-cypermethrin, deltamethrin, lambda-cyhalothrin, and permethrin, respectively.

**Table 1:** The observed visual stability, droplet size, polydispersity index (PDI), zeta potential, dynamic (absolute) viscosity, and pH of prepared pyrethroid nanoemulsions

Insecticide	Visual appearance	Droplet size (nm) ± SE	Polydispersity index (PDI) ± SE	Zeta potential (mV)	Viscosity (mPa.s) ± SE	pH	Stability after	
							Centrifugation at 5000 rpm	Heating-cooling cycle
Alpha-cypermethrin	Clear	90.26^b^ ± 3.78	0.337^c^ ± 0.01	− 0.603	60.15^b^ ± 0.12	8.51	**√**	×
Deltamethrin	Clear	172.00^a^ ± 34.07	0.827^a^ ± 0.10	− 0.669	74.67^a^ ± 7.86	7.84	**√**	×
Lambda-cyhalothrin	Clear	168.00^a^ ± 4.08	0.448^b^ ± 0.05	− 0.539	53.76^c^ ± 0.20	8.20	**√**	×
Permethrin	Clear	72.00^c^ ± 8.30	0.295^d^ ± 0.02	− 15.40	50.68^c^ ± 1.200	8.17	**√**	×

Different letters in the same column indicate significant differences according to the Student-Newman-Keuls (SNK) test (*P* ≤ 0.05). (√) refer to the stable state, (×) refer to the non-stable state. Preparation condition: 0.5 % a.i, 44% solvent (DMSO), 15% surfactant (tween 80) and 40.5% water with sonication pulses 9 cycle/s at 75% power for 15 min

#### Zeta potential

The prepared nanoemulsions revealed negative values of zeta potential (− 0.603, − 0.669, − 0.539, and − 15.4 mV for alpha-cypermethrin, deltamethrin, lambda-cyhalothrin, and permethrin, respectively) (Table 1 and Figure S2).

#### Viscosity and pH

The viscosity values of alpha-cypermethrin, deltamethrin, lambda-cyhalothrin, and permethrin nanoemulsions were 60.15, 74.67, 53.76, and 50.68 mPa.s, respectively (Table 1). The pH measurements were 8.51, 7.84, 8.20, and 8.17 for alpha-cypermethrin, deltamethrin, lambda-cyhalothrin, and permethrin, respectively.

#### Thermodynamic stability studies

The stability results after the centrifugation and heating-cooling cycle are presented in Table 1. The results showed that all nanoemulsions were transparent and stable at 5000 rpm of centrifugation and 25 °C for up to 3 months (Figure S3), while these products were separated under a heating-cooling cycle test.

#### Transmission electron microscopy

**Fig. 3:**
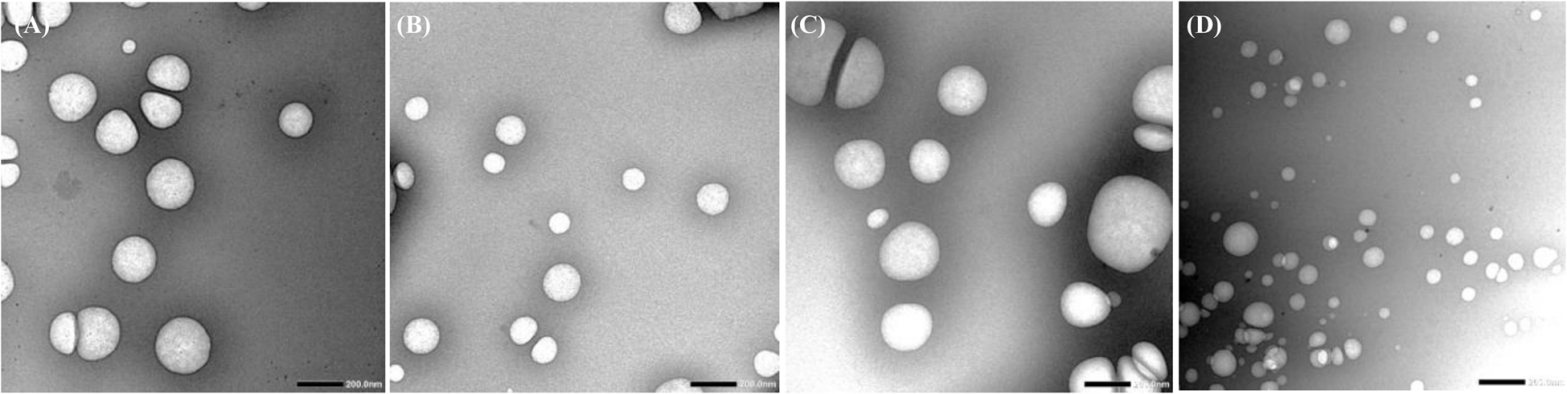
Transmission electron micrograph of prepared pyrethroid nanoemulsions alpha-cypermethrin (**A**), deltamethrin (**B**), lambda-cyhalothrin (**C**) permethrin (**D**): The TEM was performed on a JEOL JEM-1400 Plus, transmission electron microscope operating at an acceleration voltage of 80.0 kV with a 20-mm aperture. Print magnification 20,000×

The morphological study of the structure of pyrethroid nanoemulsions was carried out by TEM. Figure 3 shows the TEM micrograph of pyrethroid nanoemulsions, demonstrating the spherical shape. The droplets had a uniform shape and size. TEM analyses also confirmed the nanometric droplet diameter of formulated pyrethroids at magnification 20,000×.

### Pyrethroids released from nanoemulsions

**Fig. 4:**
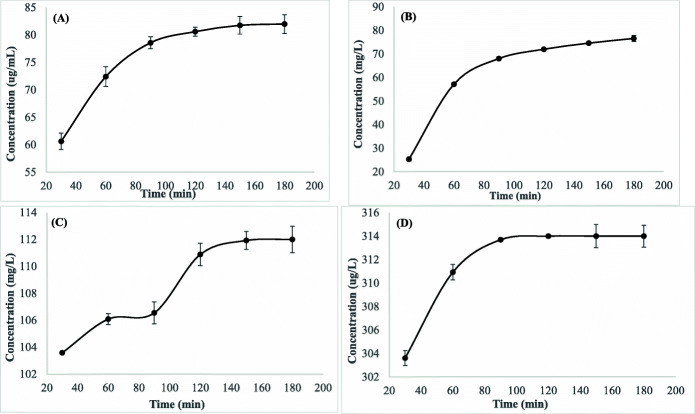
Release concentration of alpha-cypermethrin (**A**), deltamethrin (**B**), lambda-cyhalothrin (**C**), and permethrin (**D**) after time intervals for 3 h and standard error (n = 3)

The release profile assay was carried out using in vitro dialysis experiment. Cumulative amounts (mg/L) of the tested pyrethroids released from their nanoemulsions into phosphate buffer solution per time are shown in Fig. 4. Initial burst release was measured after 30 min, and the concentrations 60.60, 25.29, 103.58, and 303.60 mg/L were quantified for alpha-cypermethrin, deltamethrin, lambda-cyhalothrin, and permethrin, respectively. It was noted that the rate of permethrin released from the nanoemulsion (60%) was greater than lambda-cyhalothrin (20%), alpha-cypermethrin (12%), and deltamethrin (5%) after 30 min of the dialysis. After 180 min of the experiment, each compound’s release concentration slightly increased to 82, 112, and 314 mg/L for alpha-cypermethrin, lambda-cyhalothrin, and permethrin, respectively, whereas the concentration released from deltamethrin nanoemulsion reached only 76.58 mg/L (15%) after 180 min of the experiment.

### Larvicidal efficacy of pyrethroid nanoemulsions

The larvicidal activity of the technical, EC, and nanoemulsion of each insecticide was evaluated against *C. pipiens* larvae to record the mortality after 24 and 48 h of the exposure. The data are presented as LC_50_ values and other statistical parameters in Table 2. The results showed that alpha-cypermethrin nanoemulsion gave the LC_50_ value of 20 μg/L that was more significant than the EC formulation (40 μg/L) and the technical form (43 μg/L) after 24 h of the experiment. The technical form of deltamethrin proved an LC_50_ value of 28 μg/L, while the EC and nanoemulsion gave the LC_50_ of 26 and 28 μg/L, respectively, after 24 h. Lambda-cyhalothrin technical, EC, and nanoemulsion gave LC_50_ values 27, 23, and 15 μg/L after 24 h and 18, 13, and 10 μg/L after 48 h, respectively. However, according to the 95% confidence limit based on the probit analysis, there is no significant difference between lambda-cyhalothrin and deltamethrin. The LC_50_ values at 24 h of both insecticides interfere with the lower and upper 95% confidence limits. However, permethrin nanoemulsion proved the lowest toxicity with LC_50_ values 233 and 127 μg/L after 24 and 48 h, respectively. In permethrin, the LC_50_ values of EC and technical forms were 280 and 322 μg/L after 24 h, respectively, while the nanoemulsion was the most active form (LC_50_ = 233 and 127 μg/L after 24 and 48, respectively).

**Table 2:** Larvicidal activity of the technical, EC, and nanoemulsion of alpha-cypermethrin, deltamethrin, lambda-cyhalothrin, and permethrin against *C. pipiens* larvae

Insecticide	Time of exposure (h)	Type of formulation	LC_**50**_^**a**^ (μg/L)	95% confidence limits (μg/L)		Slope^**b**^ ± SE	Intercept^**c**^ ± SE	(χ^**2**^)^**d**^
				Lower	Upper			
Alpha-cypermethrin	24	T	43	26	46	2.47 ± 0.15	3.66 ± 0.26	20.69
		EC	40	35	64	3.22 ± 0.18	4.31 ± 0.27	35.35
		NE	20	15	26	2.30 ± 0.14	3.94 ± 0.26	22.45
	48	T	40	26	46	2.47 ± 0.15	3.66 ± 0.26	20.69
		EC	38	32	63	3.20 ± 0.18	4.21 ± 0.27	35.32
		NE	18	15	24	2.51 ± 0.15	4.37 ± 0.28	20.59
Deltamethrin	24	T	28	23	36	2.23 ± 0.13	3.45 ± 0.22	21.02
		EC	26	20	31	1.31 ± 0.08	1.52 ± 0.08	73.20
		NE	16	12	21	2.52 ± 0.16	4.50 ± 0.27	43.97
	48	T	25	19	34	2.12 ± 0.13	3.39 ± 0.21	29.62
		EC	24	20	28	1.30 ± 0.08	1.57 ± 0.09	57.09
		NE	13	9	16	2.51 ± 0.17	4.76 ± 0.30	35.17
Lambda-cyhalothrin	24	T	27	17	45	1.47 ± 0.09	2.31 ± 0.17	30.16
		EC	23	12	41	1.25 ± 0.08	2.06 ± 0.14	37.22
		NE	15	11	20	2.05 ± 0.12	3.75 ± 0.23	18.42
	48	T	18	12	27	1.30 ± 0.09	2.27 ± 0.16	18.84
		EC	13	8	18	1.43 ± 0.07	2.72 ± 0.17	17.77
		NE	10	7	14	1.42 ± 0.09	2.85 ± 0.18	19.7
Permethrin	24	T	322	221	506	1.36 ± 0.089	0.67 ± 0.081	22.655
		EC	280	206	392	1.64 ± 0.103	0.91 ± 0.09	20.66
		NE	233	201	270	1.68 ± 0.10	1.06 ± 0.09	07.20
	48	T	225	134	423	1.26 ± 0.08	0.81 ± 0.08	40.21
		EC	196	134	288	1.97 ± 0.12	1.39 ± 0.10	37.04
		NE	127	76	215	1.43 ± 0.08	1.29 ± 0.09	44.54

*T* technical, *EC* emulsifiable concentrate, *NE* nanoemulsion. ^a^The Median lethal concentration. The LC_50_ value of each compound between the other compound’s confidence limits is not significantly different. However, if the fit confidence intervals (95%) are non-overlapping, there is a significant difference between the compounds. ^b^Slope of the concentration mortality regression line ± error (SE). ^c^Intercept of the regression line ± SE. ^d^Chi-squared value

### Enzymatic activity

The data are shown in Table 3, as OD mg protein^−1^ min. The untreated larvae have 1.40, 3.31, and 2.70 for ATPase, CaE, and GST, respectively. By estimating the level of ATPase, CaE, and GST, it was found that the insecticides in nanometric formulas had a significant effect as compared to control, technical form, and EC treatment. The data proved that the activity of all tested enzymes was significantly increased except ATPase. The insecticides caused a significant ATPase inhibition up to 0.50 OD mg protein^−1^ min compared to 1.40 in control. The most effective compound on the ATPase was permethrin with specific activities of 0.50, 0.64, and 0.83 for nanoemulsion, technical, and EC, respectively. However, the lowest effective compound was alpha-cypermethrin, with activities of 0.86, 1.38, and 1.18 for technical, commercial EC, and nanoemulsion, respectively. Deltamethrin inhibited the ATPase to 0.69, 0.77, and 0.75 for the technical, EC, and nanoemulsion, respectively. Lambda-cyhalothrin gave a specific activity of 0.62 for the technical and 1.03 for the nanoemulsion.

**Table 3:** Biochemical effects of tested pyrethroids on some enzymes activity in *C. pipiens* larvae after 24 h of the treatment with LC_50_ of each compound

Treatment	Type of formulation	Specific activity (OD mg protein^−**1**^ min) ± SE		
		ATPase	CaE	GST
Untreated sample	–	1.40 ± 0.04	3.31 ± 0.01	2.70 ± 0.27
Alpha-cypermethrin	T	0.86 ± 0.02	5.06 ± 0.15	5.31 ± 0.70
	EC	1.38 ± 0.01	7.02 ± 0.50	7.54 ± 0.88
	NE	1.18 ± 0.00	4.53 ± 0.25	11.94 ± 0.84
Deltamethrin	T	0.69 ± 0.02	3.44 ± 0.19	5.49 ± 0.60
	EC	0.77 ± 0.04	3.63 ± 0.71	5.63 ± 0.80
	NE	0.75 ± 0.03	4.12 ± 0.09	5.90 ± 0.30
Lambda-cyhalothrin	T	0.62 ± 0.05	1.51 ± 0.09	4.57 ± 0.48
	EC	1.19 ± 0.02	3.32 ± 0.10	6.55 ± 0.85
	NE	1.03 ± 0.01	3.35 ± 0.01	6.15 ± 0.45
Permethrin	T	0.64 ± 0.00	2.31 ± 0.09	6.32 ± 0.59
	EC	0.83 ± 0.13	2.66 ± 0.08	7.85 ± 0.44
	NE	0.50 ± 0.01	3.37 ± 0.12	7.86 ± 0.37

*T* technical, *EC* emulsifiable concentrate, *NE* nanoemulsion, *OD* optical density, *SE* standard error, *ATPase* adenosine triphosphatase, *CaE* carboxylesterase, *GST* glutathione-S-transferase

All tested pyrethroids caused activation of the CaE, which ranged from 3.32 to 7.02 compared to 3.31 in the untreated larvae. Alpha-cypermethrin nanoemulsion was the most active with a specific activity of 7.02. It was followed by deltamethrin with specific activities of 4.53, 3.44, and 3.63 for the technical, EC nanoemulsion, respectively.

For the GST activity, permethrin was the most effective insecticide in activating this enzyme with the specific activities of 6.32, 7.85, and 7.86 for the technical, commercial EC, and nanoemulsion, respectively, compared to 2.70 in control. It was followed by alpha-cypermethrin that caused the specific activity of 5.31, 7.54, and 11.94 for the technical, EC, and nanoemulsion, respectively. The specific activity of GST treated with deltamethrin was higher than 5 for the three products. Lambda-cyhalothrin gave activity of 4.57 for the technical, 6.55 for the EC, and 6.15 for the nanoemulsion.

### Molecular docking

The docking scores and binding mechanism include H-bonds, Van der Waals, and hydrophobic interactions of the tested pyrethroids with ATPase (4BYG), CaE (5W1U), and GST (5FT3) are shown in Tables 4, 5, and 6, respectively. Analysis of the docking results showed that the pyrethroids showed a higher binding affinity with CaE and GST than ATPase. The docking scores with ATPase were ranged from − 4.33 to − 5.46 kcal/mol (Table 4). The results revealed that all insecticides exhibited H-bonding with amino acids in the active pockets of ATPase. α-Cypermethrin, deltamethrin, and permethrin exhibited H-bonding with amino acid Asn *A*112 by distances 3.54, 3.26, and 3.21 Å, respectively. Simultaneously, lambda-cyhalothrin exhibited H-bonding with Asn *A*112-N18 and Trp *A*116-N18 with 3.38 and 3.56 Å, respectively. The binding confirmation of the tested pyrethroids with ATPase is shown in Figure S4. α-Cypermethrin (Figure S4A) and deltamethrin (Figure S4C) interacted with ATPase by van der Waals (Glu 181, Gly 182, Leu 168, Pro 170, Trp 116, Val 167, and Val 183) and (Gly 113, Gly 171, Gly 182, Leu 168, Pro 170, Trp 116, Trp 169, and Val 167), respectively. Both compounds interacted by H-arene bond with amino acid Asn A112 with 3.59 and 4.17 Å, respectively. In contrast, lambda-cyhalothrin and permethrin interacted with ATPase by van der Waals (Glu 181, Gly 182, Leu 168, Pro 170, and Val 167) and (Gly 113, Phe 108, Leu 168, and Trp 116), respectively (Figure S4B and D).

**Table 4:** Molecular docking, binding scores and binding interactions of tested pyrethroids within the active sites of ATPase (PDB ID: 4BYG)

Insecticide	Docking score (S) ΔG (kcal/mol)	van der Waals	H-bond			Hydrophobic interactions (π-interactions)			RMSD
			Amino acid-ligand atom	Interaction	Distance (Å)	Amino acid-ligand atom	Interaction	Distance (Å)	
Alpha-cypermethrin	− 4.85	Glu 181, Gly 182, Leu 168, Pro 170, Trp 116, Val 167, Val 183	Asn *A*112*-*N15	HBA	3.54	Asn *A*112-6-ring	Arene-H	3.59	1.66
Lambda-cyhalothrin	− 5.46	Glu 181, Gly 182, Leu 168, Pro 170, Val 167	Asn *A*112*-*N18 TRP *A*116-N18	HBA HBA	3.38 3.56	–	–	–	1.88
Deltamethrin	− 4.33	Gly 113, Gly 171, Gly 182, Leu 168, Pro 170, Trp 116, Trp 169, Val 167	Asn *A*112*-*N15	HBA	3.26	Asn *A*112-6-ring	Arene-H	4.17	1.96
Permethrin	− 4.61	Gly 113, Phe 108, Leu 168, Trp 116	Asn *A*112*-*O1	HBA	3.21	-	-	-	3.36

*RMSD* the root mean square deviation of the pose in Å, from the original ligand. This field is present if the site definition was identical to the ligand definition. Residues/water molecules participating in hydrogen bonds and close van der Waals contacts (< 4 Å) with the inhibitors

**Table 5:** Molecular docking, binding scores and binding interactions of tested pyrethroids within the active sites of CaE (PDB ID: 5W1U)

Insecticide	Docking score (S) ΔG (kcal/mol)	van der Waals	H-bond			Hydrophobic interactions (π-interactions)			RMSD
			Amino acid-ligand atom	Interaction	Distance (Å)	Amino acid-ligand atom	Interaction	Distance (Å)	
Alpha-cypermethrin	− 9.35	Arg 73, Arg 392, Asp 279, Glu 118, Gly 109, Gly 110, Gln 330 His 442, Leu 327, Leu 328, Lys 331, Phe 281 Ser 191, Trp 224, Tyr 428, Val 393	Asp 279*-*Cl11 Leu 328-N15	HBD HBA	3.32 0.6	–	–	–	1.14
Lambda-cyhalothrin	− 10.01	Arg 73, Arg 74, Asn 452, Ala 443, Gln 330, Glu 113, Gly 109, Gly 110, His 427, His 442, Leu 120, Leu 327, Leu 446, Met 432, Phe 281, Ser 191, Ser 447, Thr 112, Tyr 121, Tyr 428, Phe 281	Glu 113*-*C16 Arg 73-F11	HBD HBA	2.90 2.90	–	–	–	2.03
Deltamethrin	− 8.72	Arg 73, Glu 113, Glu 268, Gly 109, Gly 110, Gln 330, His 442, Leu 327, Leu 334, Lys 331, Phe 281, Ser 191, Tyr 428	Ser 191*-*Br8 His 442-Br8 Arg 73-O1	HBD HBD HBA	3.46 3.62 2.95	–	–	–	2.21
Permethrin	− 7.44	Arg 73, Gln 330, Glu 113, Glu 268 Gly 109, Leu 328, Leu 328, Lys 331, Phe 281, Thr 112	Lys 331*–*O1	HBA	3.04	Arg 73-6-ring	Pi-cation	3.90	1.93

*RMSD* the root mean square deviation of the pose in Å, from the original ligand. This field is present if the site definition was identical to the ligand definition. Residues/water molecules are participating in hydrogen bonds and close van der Waals contacts (< 4 Å) with the inhibitors

**Table 6:** Molecular docking, binding scores and binding interactions of tested pyrethroids within the active sites of GST (PDB ID: 5FT3)

Insecticide	Docking score (S) ΔG (kcal/mol)	van der Waals	H-bond			Hydrophobic interactions (π-interactions)			RMSD
			Amino acid-ligand atom	Interaction	Distance (Å)	Amino acid-ligand atom	Interaction	Distance (Å)	
Alpha-cypermethrin	− 8.55	Arg 112, Glu 116, His 41, Leu 42, Leu 111, Leu 119, Lys 39, Phe 108, Phe 120,Pro 13,Thr 54, Val 11, Val 55, Val 207	Leu *A*36*-*Cl11 His 53-N15	HBD HBA	3.56 2.58	–	–	–	1.66
Lambda-cyhalothrin	− 9.95	Arg *A*112, Cys *A*115, Glu A116, His *A*41, His *A*53, Leu *A*36, Leu *A*111, Leu *A*119, Lys *A*39, Lys *B*136, Phe *A*108, Pro *A*13, Ser *A*12, Val *A*11, Val *A*207	Thr *A54-*N18 Val *A*55-N18	HBA HBA	3.19 3.53	Phe *A*120-6-ring	Arene-H	4.06	1.50
Deltamethrin	− 8.24	Glu *A*116, Leu *A*36, Leu *A*42, Leu *A*111, Lys *A*39, Phe *A*120, Phe *A*108, Pro *A*13, Thr *A*54, Val *A*55	His *A*41-O1 His A53-N15 Arg A112-N15	HBA HBA HBA	3.09 3.68 2.76	ASN *A*112-6-ring	Arene-H	4.17	0.58
Permethrin	− 8.53	Arg 112, Cys 115, Glu 116, His 53, Leu 36, Leu 111, Leu 119, Phe 108, Phe 120, Pro 13, Thr 54, Val 11, Val 55, Val 207	Ser *A*12*-*O1	HBA	3.24	–	–	–	1.76

*RMSD* the root mean square deviation of the pose in Å, from the original ligand. This field is present if the site definition was identical to the ligand definition. Residues/water molecules are participating in hydrogen bonds and close van der Waals contacts (< 4 Å) with the inhibitors

Tested pyrethroids exhibited binding affinity ranged from − 7.44 to − 10.01 kcal/mol on the active sites of CaE (Table 5). Lambda-cyhalothrin was the highest (ΔG = − 10.01 kcal/mol) followed by α-cypermethrin, deltamethrin, and then permethrin ΔG values of − 9.35, − 8.72, and − 7.44 kcal/mol, respectively. Alpha-cypermethrin interacted with the CaE enzyme through two hydrogen bonds (Asp 279*-*CL11 and Leu 328-N15) with distances of 3.32 and 0.6 Å, respectively. Besides, some van der Waals bonds ( Arg 73, Arg 392, Asp 279, Glu 118, Gly 109, Gly 110, Gln 330 His 442, Leu 327, Leu 328, Lys 331, Phe 281 Ser 191, Trp 224, Tyr 428, and Val 393) are included (Figure S5A). Lambda-cyhalothrin bonded through HBD, HBA (Arg 73-F11 and Glu 113-C16, respectively) with 2.9 Å for both and van der Waals interactions (Arg 73, Arg 74, Asn 452, Ala 443, Gln 330, Glu 113, Gly 109, Gly 110, His 427, His 442, Leu 120, Leu 327, Leu 446, Met 432, Phe 281, Ser 191, Ser 447, Thr 112, Tyr 121, Tyr 428, and Phe 281) (Figure S5B). Three hydrogen bonds (Ser 191*-*Br8, His 442-Br8, and Arg 73-O1) with distances of 3.46, 3.62, and 2.95 Å, respectively, and thirteen van der Waals (Arg 73, Glu 113, Glu 268, Gly 109, Gly 110, Gln 330, His 442, Leu 327, Leu 334, Lys 331, Phe 281, Ser 191, and Tyr 428) were formed between deltamethrin and CaE (Figure S5C). Figure S5D shows the interactions between permethrin and CaE, which was through one HBA (Lys 331*–*O1), ten van der Waals (Arg 73, Gln 330, Glu 113, Glu 268 Gly 109, Leu 328, Leu 328, Lys 331, Phe 281, and Thr 112) and Pi-cation interaction (Arg 73-6-ring, 3.90 Å).

The docking results with GST (Table 6) indicated that lambda-cyhalothrin was the highest affinity binding with the lowest energy value − 9.95 kcal/mol. It was followed by alpha-cypermethrin, permethrin, deltamethrin with energy values − 8.55, − 8.53, and − 8.24 kcal/mol. alpha-cypermethrin interacted with GST through van der Waals with 14 amino acids (Arg 112, Glu 116, His 41, Leu 42, Leu 111, Leu 119, Lys 39, Phe 108, Phe 120, Pro 13, Thr 54, Val 11, Val 55, and Val 207) with docking score of − 8.55 kcal/mol (Figure S6A). Figure S6B shows the interactions between lambda-cyhalothrin and GST through van der Waals with 15 amino acids (Arg *A*112, Cys *A*115, Glu A116, His *A*41, His *A*53, Leu *A*36, Leu *A*111, Leu *A*119, Lys *A*39, Lys *B*136, Phe *A*108, Pro *A*13, Ser *A*12, Val *A*11, and Val *A*207) and arene H-bond with Phe *A*120. Deltamethrin reacted with GST through 10 van der Waals (Glu *A*116, Leu *A*36, Leu *A*42, Leu *A*111, Lys *A*39, Phe *A*120, Phe *A*108, Pro *A*13, Thr *A*54, and Val *A*55), 3 H-bonds with amino acids ( His *A*41, His *A*53, and Arg *A*112 ) and H-arene bond with amino acid ASN *A*112 (Figure S6C). Permethrin interacted with the pocket of GST through H-bond with amino acid Ser *A*12 and 14 amino acids through van der Waals bonds (Arg 112, Cys 115, Glu 116, His 53, Leu 36, Leu 111, Leu 119, Phe 108, Phe 120, Pro 13, Thr 54, Val 11, Val 55, and Val 207) (Figure S6D).

### Ecotoxicity study against the freshwater green alga

The toxicity endpoint values after acute exposure of *R. subcapitata* to different forms of pyrethroids used as mosquito larvicides are illustrated in Table 7. The sensitivity of *R. subcapitata* to insecticides; expressed as EC_50_, ranged from 0.76 to > 100 mg/L. Based on these values, the decreasing order of the sensitivity was commercial EC > NE > technical form. The current data disclosed that the commercial EC of the tested insecticides were more toxic to *R. subcapitata* and recorded 0.76, 4.92, 5.03, and 16.98 mg/L for deltamethrin, permethrin, lambda-cyhalothrin, and alpha-cypermethrin, respectively. The potency of commercial EC may be attributed to the additives in the formulation rather than the active ingredient.

**Table 7:** EC_50_ and NOEC (mg/L) of nanoformulations compared with technical and commercial formulated pyrethroids to freshwater microalga *R. subcapitata*. The 95% confidence limits of the EC_50_ values are indicated in parentheses

Insecticide	Formulation	Toxicity endpoint		
		96 h EC_**50**_ (mg/L)	NOEC (mg/L)	GHS hazard statement
Alpha-cypermethrin	T	69.33 (38.15–145.54)	6.932	H402
	EC	16.98 (10.99–29.01)	1.698	H402
	NE	101.11 (38.08–409.14	1.011	S
Lambda-cyhalothrin	T	33.89 (15.93–94.27)	3.389	H402
	EC	5.03 (2.96–9.62)	0.503	H401
	NE	11.29 (1.83–121.71)	1.129	H402
Deltamethrin	T	> 100	> 10	S
	EC	0.76 (0.56–1.05)	0.076	H400
	NE	13.14 (6.49–30.74)	1.314	H402
Permethrin	T	14.94 (9.72–23.72)	1.494	H402
	EC	4.92 (3.31–7.75)	0.492	H401
	NE	20.55 (12.66–36.25)	2.055	H402

*T* technical, *EC* emulsifiable concentrate, *NE* nanoemulsion, *NOEC* no observed effect concentration on algal growth rate, *H* hazard statement. *H400* very toxic to aquatic life (hazardous to the aquatic environment, acute hazard, category 1; ≤ 1mg/L); *H401* toxic to aquatic life (hazardous to the aquatic environment, acute hazard, category 2; > 1–≤ 10 mg/L); *H402* harmful to aquatic life (hazardous to the aquatic environment, acute hazard, category 2; > 10–≤ 100 mg/L). S: Safe use (no hazard statement is suggested) since acute toxicity > 100 mg/L

Toxicity of nanoformulations showed a different pattern where alpha-cypermethrin exhibited a safe effect on *R. subcapitata* (EC_50_ > 100 mg/L) while the EC_50_ for the other insecticides recorded 11.29, 13.14, and 20.55 mg/L for lambda-cyhalothrin, deltamethrin, and permethrin, respectively. The sensitivity of *R. subcapitatata* towards nanoformulations was lambda-cyhalothrin > deltamethrin > permethrin. The differential toxicity of nanoformulations depends on their nanostructure and high surface to mass ratio as well as the nature of their constitutive element. On the other hand, the EC_50_ values for the technical form of the tested insecticides were 33.89, 69.33, >100, and 14.94 mg/L for lambda-cyhalothrin, cypermethrin, deltamethrin, and permethrin, respectively. The sensitivity of *R. subcapitata* was permethrin > lambda-cyhalothrin > cypermethrin > deltamethrin.

For subsequent characterization of the potentially hazardous effects of the tested insecticides and addressing safety issues of the developed nanopesticides, both NOEC and hazard statements were evaluated (Table 7). The data showed that all nanoformulations represent category acute III with harmful effects to aquatic life (H402) compared with the commercial EC forms which represent category acute I and II with very toxic and/or toxic hazardous effects to the aquatic life (H400 and H410).

## Discussion

### Physicochemical properties of the tested pyrethroids

According to Lipinski’s “rule of five” [35], good intestinal absorption and oral bioavailability of compounds reflect RB and MR’s acceptable values. The stereo-specificity of the drug molecule is a property of nRB, which was found to be < 10. There are no hydrogen bond donors (HBD) in the tested pyrethroids, while the number of hydrogen bond acceptors (HBA) ranged from 3 to 7. The literature has also documented that excellent absorption in the intestine is induced by PSA < 140 [36]. The Log S value for all insecticides is between − 6.84 and − 7.22, indicating low water solubility.

### Characterizations of the pyrethroid nanoemulsions

Several studies prepared and characterized pyrethroid nanoemulsions, such as alpha-cypermethrin, deltamethrin, lambda-cyhalothrin, and permethrin [13, 14]. The droplet size of the prepared nanoemulsions is in agreement with other studies. Mishra and others reported that nano-sized permethrin’s mean particle size was 175.3 nm [13], whereas the TEM analysis investigated by Patel et al. [37] revealed that cypermethrin particle size’s encapsulation was ranged between 115 and 119 nm. However, the droplet size of beta cypermethrin nanosuspension prepared by Zeng et al. [38] was 168 nm. It was observed no phase separation, creaming, and sedimentation under room temperature (25 °C) and accelerated stability evaluation [8]. The long-term physical stability of a nanoemulsion related to its small droplets makes this type of formulation being referred to as “approaching thermodynamic stability.” The average droplet size of the nanoemulsions typically falls within the range of 20–500 nm [19]. The small size of the droplets in nanoemulsions gives them some advantages over conventional emulsions. These advantages include higher optical clarity, higher stability to droplet aggregation and gravitational separation, and higher bioactivity of encapsulated components. The nanoemulsions have emerged as alternative drug carriers because they increase the dissolution rates and bioavailability of many poorly soluble drugs in water [9].

PDI reflects the distribution of the particle size in a formulation. The PDI is a dimensionless measure of the width of size distribution calculated from the cumulated analysis and ranges from zero to one [39]. A lower PDI value (near zero) indicates the existence of a uniform distribution of droplet size and homogenous populations, whereas a PDI value closer to 1 (one) displays a wide range of droplet sizes (heterogeneity of the system). The PDI value around 0.2 indicates the droplet population’s homogeneity in prepared formulations. Besides other important criteria, zeta potential is another essential characteristic of the nanoemulsions and an indicator of the nanoemulsion stability associated with the droplets’ surface potential. The negative values are necessary for droplet-droplet repulsion and thus enhanced nanoemulsion stability [40]. The high stability of formulations with zeta potential values is associated with repulsive forces that exceed attracting van der Waals forces, resulting in dispersed particles and a deflocculated system. The range of pH value of nanoemulsion has a strong effect on its stability. The different pH value levels lead to a change in the globules’ surface charge and their stability during storage. Keeping different nanoemulsions under environmental storage conditions may be an essential criterion for judging effectiveness, potency, and stability [19].

### Release studies of pyrethroid nanoemulsions

The efficiency of nanoformulation to extend residence time, reduce insecticide losses, and reduce overuse. It also makes the pesticide’s continuous and stable release possible [8]. Our results agree with the results obtained by Nguyen et al. [41], who proved that the release rate of deltamethrin nanoemulsion was lower than 20% in the first 3 h of the experiment. In addition, it confirmed that lambda-cyhalothrin /polyurethane nanoemulsion had a slower release rate than the traditional formulations. In addition, the release profile of the lambda-cyhalothrin-loaded nanoemulsion was compared to its EC and WP formulations at 25 °C [8]. The results reported that the lambda-cyhalothrin released from EC and WP was very fast and reached equilibrium after 48 h, and the accumulated releases were over 90%. However, the release rate of lambda-cyhalothrin nanoemulsion was rapid within the first 30 h, and then, it slowed down and maintained a stable release until equilibrium after 80 h.

### Toxicity against *C. pipiens* larvae

The effect of the three forms of the tested insecticides was significantly different against *C. pipiens* larvae. It can be noted that the technical form exhibited the lowest larvicidal activity. However, the EC of all tested insecticides slightly improved the toxic action against the larvae. However, all insecticides’ nanoemulsions showed significantly high toxicity (1.5–2-fold) compared to the technical and EC. This finding led to a significant decrease in the field application rate by half-value, resulting in low environmental pollution and hazards.

The nanoscale form of pesticides has been applied to control the developed resistance in insect species, attributed to conventional pesticides’ excessive use. Compared to the traditional pesticides, the higher efficacy of nano pesticides was observed. In agreement with our results, other studies proved that pyrethroids’ preparation in nanoemulsion form made them more active than the conventional forms [9, 14]. Mishra et al. [13] prepared nano-sized permethrin in its colloidal state and studied its effect on *C. tritaeniorhynchus* larvae. They found that the LC_50_ of the bulk permethrin was 442 μg/L. In contrast, the LC_50_ of the nano-permethrin was 57 μg/L. The present study also supports nano pesticides’ ability to control mosquito vectors. Reducing nanoemulsions and elevating their surface area could facilitate their passive penetration into the target pest, thus enhancing their toxicity [13]. As the results presented, alpha-cypermethrin, deltamethrin, and lambda-cyhalothrin were the most toxic insecticides (LC_50_ ranged from 10 to 43 μg/L) compared to the permethrin (LC_50_ ranged from 127 to 322 μg/L) against *C. pipiens* larvae. This finding refers to the pyrethroid type’s chemical structure that the alpha-cypermethrin, deltamethrin, and lambda-cyhalothrin are cyano-derivatives. However, permethrin is a non-cyano-derivative. As well-known from the literature, cyano-derivatives of the pyrethroids were more active against different pests than the non-cyano derivatives [42].

### Biochemical studies

To elucidate some biochemical actions of the tested pyrethroids on *C. pipiens* larvae, the effect of the LC_50_ values on the ATPase, CaE, and GST isolated from the survived treated larvae after 24 h was examined. In agreement with our findings, Kakko et al. [43] proved that cypermethrin was the most toxic against ATPase, followed by permethrin and natural pyrethrin. The cell toxicity was dependent on the chemical structure of pyrethroids. The pyrethroids without the α-cyano group show the weakest physiological effect. Clark and Matsumura [44] measured Na^+^-Ca^++^ ATP hydrolysis and Ca-Mg ATP hydrolysis in cockroach brain tissue under in vitro conditions. They found that the non-cyano-containing pyrethroids inhibited Na^+^-Ca^++^-ATP hydrolysis mostly than their cyano-containing counterparts. The reverse is true for pyrethroid action on Ca^++^-Mg^++^-ATP hydrolysis.

As well known, CaEs hydrolyze numerous endogenous and exogenous ester-containing compounds. Therefore, they play a vital role in the detoxification of pyrethroids, strongly related to the resistance phenomenon. Identification of CaE genes associated with pyrethroid resistance was investigated in the malaria vector *Anopheles sinensis* [45] and the mosquito *Aedes aegypti* [46].

In disagreement with our results, Kostaropoulos et al. [47] proved that the pyrethroids bind with the active site of GST, resulting in a significant decrease of its activity towards CDNB in a competitive manner, but was not conjugated with GSH. Grant and Matsumura [48] found a variation in the action and level of GST due to its interaction with pyrethroids, studied GST as an antioxidant defense agent confer pyrethroid resistance in *Nilaparvata lugens* and demonstrated that lambda-cyhalothrin and permethrin induced oxidative stress and lipid peroxidation in insects. For these reasons, they hypothesized that the prominent role of elevated GSTs in conferring resistance in *N. lugens* is through protecting tissues from oxidative damage. Markus et al. also elucidated the inhibitory activity of deltamethrin against human GST [49]. They proved that deltamethrin was a potent inhibitor of GST-P1-1, and it inhibited the homodimeric enzyme in a non-competitive manner. Thus, the purpose of determining ATPase, CaE, and GST levels as essential parameters to study the toxic effect of nano-pesticides on insect vector species.

### Docking studies

It is well known that molecular docking is a method to predict and understand molecular recognition, find the predominant binding mode and binding affinity between the protein and ligand, and give a three-dimensional structural explanation of the protein-ligand interaction. The bond interactions were useful for elucidation of several biological activities of tested compounds as larvicides [50]. Zeng et al. studied the interactions of pepsin with deltamethrin and cyhalothrin by multi-spectroscopic approaches and molecular docking [50]. They approved that the tested pyrethroids bounded directly into the enzyme cavity site. The binding was influenced by the active site’s microenvironment resulting in the extension of peptide strands with loss of α-helix structures. Kumar et al. illustrated the molecular interactions of some pyrethroids including cypermethrin towards adaptive immune cell receptors of T (CD4 and CD8) and B (CD28 and CD45) [51]. They found that fenvalerate (− 5.534 kcal/mol: CD8), fluvalinate (− 4.644 and − 4.431 kcal/mol: CD4 and CD45), and cypermethrin (− 3.535 kcal/mol: CD28). Data exhibited less docking energy or more affinity for B cell and T cell immune receptors, which may later result in immunosuppressive and hypersensitivity reactions. Markus et al also elucidated the inhibitory activity of deltamethrin against human GST [49]. They found that deltamethrin appears to fit well in an eccentric cavity located at the GST homodimer, likely causing conformational changes at the enzyme’s substrate binding sites such that the enzyme is no longer able to effectively convert GSH and CDNB.

### Biosafety evaluation against the freshwater green alga

Treating the aquatic environment with nanomaterials to control mosquito larvae or other pests may lead to important risks for non-target aquatic organisms [52]. Both physicochemical and toxicological properties of nanomaterials would permit and control environmental risk assessment and safety of these materials [53]. Microalgae are widely used in bioassay toxicity testing of aquatic pollutants since they are sensitive organisms with a high capacity of bioaccumulation due to their high surface of contact [54].

A concentration-response ratio established for *R. subcapitata* and 96 h EC_50_ values are shown in Table 7. Considering the values obtained for EC_50_, it was observed that this organism was more sensitive and highly affected by the commercial form (EC) of all tested insecticides after acute exposure, followed by technical form and/or nanoformulations. It is worthy to mention that the synthesized nanoformulation are readily soluble in water with no agglomeration and proved to be safe to algae and aquatic organisms when tested as alpha-cypermethrin nanoemulsion and less toxic (2–17-fold) than the commercial EC in case of lambda-cyhalothrin, deltamethrin and permethrin nanoemulsions.

Similar results were obtained by Grillo et al. [55] who stated that paraquat-loaded chitosan nanoparticles showed less toxicity than paraquat (96 h EC_50_s were 1.15 and 0.48 mg/L; respectively). Also, other ecotoxicity studies demonstrated that thiamethoxam nanoparticles were less toxic than commercial formulations for *R. subcapitata* and non-toxic for *A. salina* under the conditions of the study. Based on the existing knowledge, the method of green synthesis of nanoparticles and several green-fabricated metal nanoparticles failed to show toxicity against different aquatic organisms. *Plumeria rubra*- and *Pergularia daemia*-synthesized Ag nanoparticles did not exhibit any evident toxicity against fishes after 48 h of exposure to concentrations corresponding to the LC_50_ and LC_90_ values on IV instar larvae of *Ae. aegypti* and *An. stephensi* [56]. Subarani et al. [57] also did not report the toxicity effects of *Catharanthus roseus*-synthesized Ag nanoparticles against fish and mosquito predators; *G. affinis* after 72 h of exposure.

As related to NOEC values among tested insecticides (Table 7), it represents the highest test concentration at which no toxic effects are observed, and went parallel to the EC_50_ pattern recorded in the current study. However, NOEC can be regarded as a chronic endpoint, and values indicated in this study reflect the concentrations that can offer minimum protection to the test organism; *R. subcapitata* against tested insecticides particularly on a long-term basis. Furthermore, classification of tested insecticides according to their potential hazard statements to the aquatic ecosystem, an only commercial form of deltamethrin can be considered highly hazardous to *R. subcapitata* (category acute I; H400) and is not recommended for application in waterways, whereas its nanoformulation exhibited a less hazardous effect on the test alga. Additionally, all the tested nanoformulations showed only harmful effects to aquatic life (category acute III; H402) compared with very toxic or toxic hazardous effects (category I or II; H 400 or 401) of commercial forms of the same insecticides to aquatic life.

It can be concluded that, for safety purposes, nanopesticides can be recommended for use in vector control programs in waterways and can be considered highly promising for the development of safe insecticides against mosquitoes. The nanopesticides are less harmful to the environment and more efficient (in terms of cost and performance) than the existing formulations. Nevertheless, only further research will show whether the research results can find their way to application in practice.

## Conclusion

Permethrin from type I (non-cyano) and three pyrethroids from type II (alpha-cypermethrin, deltamethrin, and lambda-cyhalothrin) were prepared in nanoemulsions. The modification of these compounds to nanoform increased the insecticidal properties. 0.5% a.i, 44% DMSO, 15% tween 80, 40.5% water, 9 cycle/s of sonication pulses, 75% power for 15 min were selected as the optimal conditions for preparation of the insecticide nanoemulsions. The remarkable stable behavior of prepared nanopesticides with adequate larvicidal activity at the lowest exposure concentration makes it a suitable and effective mosquito control agent. In addition, the evaluation of the biosafety of nanoscale pesticides against freshwater alga *R. subcapitata* by calculating different toxicity parameters establishes the non-toxic behavior of insecticide concentrations applied against non-target species. This confirms environmental safety with strong efficacy as a mosquitocidal agent against larvae. Also, the data proved the greatest effect of the nanoemulsions as alternatives to the conventional pesticide formulations.

## Supplementary Information

**Additional file 1: **SUPPLEMENTARY MATERIALS (DATA IN BRIEF). **Table S1**. Chemical structure and physicochemical properties of the tested pyrethroids. Description of data: This table shows the chemical structure and physicochemical properties of the tested compounds. The tested compounds' molecular weight was 416.3, 505.21, 449.85, and 505 g/mol for alpha-cypermethrin, deltamethrin, lambda-cyhalothrin, and permethrin, respectively. The polar surface area (PSA) of all tested pyrethroids was 59.32, except permethrin was 35.53,. wWhile the hydrophobicity factor (ALogP) of all tested compounds was around 6. There are no hydrogen bond donors (HBD) in the tested pyrethroids, while the number of hydrogen bond acceptors (HBA) ranged from 3 to 7. **Table S2**. HPLC gradient solvent system for separation of alpha-cypermethrin, deltamethrin, lambda-cyhalothrin and permethrin. Description of data: This table shows the HPLC conditions used for the separation of pyrethroids understudy. These conditions include the gradient solvent system and the optimum wavelength used in the separation process. **Table S3**. Experimental factorial design for preparation and optimization of deltamethrin nanoemulsions. Description of data: This table shows the different experimental setup using Minitab software was used to determine the influence of six independent variables on the pyrethroid nanoemulsions' characterization (dependent variable). In these optimization experiments, deltamethrin was selected as a model of the tested pyrethroids. **Table S4**. The observed visual stability, droplet size, polydispersity index (PDI), zeta potential, dynamic (absolute) viscosity, and pH of prepared deltamethrin nanoemulsions. Description of data: This table presents the quantitative results of nanoemulsion pyrethroids include the droplet size (nm), PDI, pH, and viscosity (mPa.s). The data proved that there are significant differences in the droplet size of the nine prepared deltamethrin formulations. In the PDI case, there are no significant differences between the formulations (0.516-0.964) except formulation 2 (PDI = 0.158). There is no significant difference between the viscosity values of formulations 1, 2, 5, and 6. However, formulations 4, 8, and 9 showed significant differences in their viscosity values. The pH of the prepared formulations was in the range of 7.78-8.18. **Figure S1**. The visual appearance of prepared deltamethrin nanoemulsions. The code number represents the experimental factorial design shown in Table 2. Description of data: This figure presents the visual appearance of the nine produced deltamethrin nanoemulsions formulations. **Figure S2**. Zeta potential distribution graph of pyrethroid nanoemulsions of alpha-cypermethrin (A), deltamethrin (B), lambda-cyhalothrin (C), and permethrin (D). Description of data: This figure presents a zeta potential distribution graph of prepared pyrethroid nanoemulsions. **Figure S3**. The visual appearance of pyrethroid nanoemulsions of alpha-cypermethrin (1), deltamethrin (2), lambda-cyhalothrin (3), and permethrin (4). Description of data: This figure presents the visual appearance of alpha-cypermethrin (1), deltamethrin (2), lambda-cyhalothrin (3), and permethrin (4) nanoemulsions. **Figure S4**. Docking view of the tested pyrethroids on the binding sites of ATPase (PDB ID: 4byg). Alpha-cypermethrin (A), lambda-cyhalothrin (B), deltamethrin, (C), and permethrin (D). Description of data: This figure presents the docking view of the tested pyrethroids on the binding sites of ATPase (PDB ID: 4byg). Left is the 2D interaction diagram structure and right is the complex structure in stereo view (3D). **Figure S5**. Docking view of the tested pyrethroids on the binding sites of CaE (PDB ID: 5w1u). Alpha-cypermethrin (A), lambda-cyhalothrin (B), deltamethrin, (C), and permethrin (D). Description of data: This figure presents the docking view of the tested pyrethroids on the binding sites of CaE (PDB ID: 5w1u). Left is the 2D interaction diagram structure, and right is the complex structure in stereo view (3D). **Figure S6**. Docking view of the tested pyrethroids on the binding sites of GST (PDB ID: 5ft3). Alpha-cypermethrin (A), lambda-cyhalothrin (B), deltamethrin, (C), and permethrin (D). Description of data: This figure presents the docking view of the tested pyrethroids on the binding sites of GST (PDB ID: 5ft3). Left is the 2D interaction diagram structure, and right is the complex structure in stereo view (3D).

## Data Availability

All data generated or analyzed during this study are included in this article. Also, the related datasets are available from the corresponding author on reasonable request.

## Abbreviations

ANOVA: Analysis of variance
a.i: Active ingredient
ATP: Adenosine triphosphate
ATPase: Adenosine triphosphatase
BSA: Bovine serum albumin
*C. pipiens*: *Culex pipiens*
CaE: Carboxylesterase
CDNB: 1-Chloro-2,4-dinitrobenzene
DLS: Dynamic light scattering
DMSO: Dimethylsulfoxide
EC: Emulsifiable concentrate
EDTA: Ethylenediaminetetraacetic acid
GSH: L-glutathione
GST: Glutathione-S-transferase
LC_50_: Median lethal concentration
MOE: Molecular Operating Environment
O/W: Oil/water
PDB: Protein data bank
PDI: Polydispersity index
SE: Standard error
SPSS: Statistical Package for the Social Sciences
TCA: Trichloroacetic acid
TEM: Transmission electron microscopy
WHO: World Health Organization
β-NAD: β-Nicotinamide adenine dinucleotide
