# Supplementary material for: Enhanced mosquitocidal efficacy of pyrethroid insecticides by nanometric emulsion preparation towards Culex pipiens larvae with biochemical and molecular docking studies
Source: J Egypt Public Health Assoc. 2021 Jul 15;96:21. doi: 10.1186/s42506-021-00082-1 (PMC8282878; doi:10.1186/s42506-021-00082-1)
Supplement: Supplementary file 1 — Additional file 1: SUPPLEMENTARY MATERIALS (DATA IN BRIEF). Table S1. Chemical structure and physicochemical properties of the tested pyrethroids. Description of data: This table shows the chemical structure and physicochemical properties of the tested compounds. The tested compounds' molecular weight was 416.3, 505.21, 449.85, and 505 g/mol for alpha-cypermethrin, deltamethrin, lambda-cyhalothrin, and permethrin, respectively. The polar surface area (PSA) of all tested pyrethroids was 59.32, except permethrin was 35.53,. wWhile the hydrophobicity factor (ALogP) of all tested compounds was around 6. There are no hydrogen bond donors (HBD) in the tested pyrethroids, while the number of hydrogen bond acceptors (HBA) ranged from 3 to 7. Table S2. HPLC gradient solvent system for separation of alpha-cypermethrin, deltamethrin, lambda-cyhalothrin and permethrin. Description of data: This table shows the HPLC conditions used for the separation of pyrethroids understudy. These conditions include the gradient solvent system and the optimum wavelength used in the separation process. Table S3. Experimental factorial design for preparation and optimization of deltamethrin nanoemulsions. Description of data: This table shows the different experimental setup using Minitab software was used to determine the influence of six independent variables on the pyrethroid nanoemulsions' characterization (dependent variable). In these optimization experiments, deltamethrin was selected as a model of the tested pyrethroids. Table S4. The observed visual stability, droplet size, polydispersity index (PDI), zeta potential, dynamic (absolute) viscosity, and pH of prepared deltamethrin nanoemulsions. Description of data: This table presents the quantitative results of nanoemulsion pyrethroids include the droplet size (nm), PDI, pH, and viscosity (mPa.s). The data proved that there are significant differences in the droplet size of the nine prepared deltamethrin formulations. In the PDI case, th [file 42506_2021_82_MOESM1_ESM.docx]

**SUPPLEMENTARY MATERIALS (DATA IN BRIEF)**

**Enhanced mosquitocidal efficacy of pyrethroid insecticides by nanometric emulsion preparation towards *Culex pipiens* larvae with biochemical and molecular docking studies**

**Nehad E. M. Taktak^1^, Mohamed E. I. Badawy^2*^, Osama M. Awad^1^, Nadia E. Abou El-Ela^1^ and Salwa M. Abdallah^3^**

*^1^Department of Tropical Health, High Institute of Public Health, 165 El-Horreya Ave., 21561-*El-Hadara*, Alexandria University, Alexandria, Egypt.*

*^2^Department of Pesticide Chemistry and Technology, Faculty of Agricul5mjbh8ture, 21545-El-Shatby, Alexandria University, Alexandria, Egypt*

*^3^ Mammalian and Aquatic Toxicology Department, Central Agricultural Pesticides Laboratory (CAPL), Agricultural Research Center (ARC), 12618-Dokki, Egypt*

**^*^** Address correspondence to **Mohamed E. I. Badawy**, Professor of Pesticide Chemistry and Toxicology, Department of Pesticide Chemistry and Technology, Laboratory of Pesticide Residues Analysis, Faculty of Agriculture, Aflatoun St., 21545-El-Shatby, Alexandria University; Alexandria, Egypt. Phone: 002039575269; Fax: 002035972780; E-mail: [m_eltaher@yahoo.com](mailto:m_eltaher@yahoo.com).

**Table S1.** Chemical structure and physicochemical properties of the tested pyrethroids

| **Insecticide** | **Chemical structure** | **MW (g/mol)** | **ALogP** | **HBA** | **HBD** | **RB** | **MR** | **PSA** | **Log S** | **Lipinski violation** |
| --- | --- | --- | --- | --- | --- | --- | --- | --- | --- | --- |
| Alpha-cypermethrin |  | 416.30 | 6.94 | 4 | 0 | 7 | 108.96 | 59.32 | -6.84 | 1 violation: LogP>5 |
| Deltamethrin |  | 505.21 | 6.20 | 4 | 0 | 7 | 115.11 | 59.32 | -7.22 | 2 violation: MW>500 and LogP>5 |
| Lambda-cyhalothrin |  | 449.85 | 6.10 | 7 | 0 | 7 | 109.17 | 59.32 | -7.08 | 1 violation: LogP>5 |
| Permethrin |  | 391.28 | 6.5 | 3 | 0 | 7 | 104.41 | 35.53 | -7.13 | 1 violation: LogP>5 |

**MW**: Molecular weight. **ALogP**: Hydrophobicity factor (octanol/water partition coefficient). **HBA**: Number of hydrogen bond acceptors. **HBD**: Number of hydrogen bond donors. **RB**: Freely Rotating Bonds. **MR**: Molar refractivity. **PSA**: Molecular polar surface area. **Log S**: log of solubility.

**Table S2.** HPLC gradient solvent system for separation of α-cypermethrin, deltamethrin, lambda-cyhalothrin and permethrin

| **Time (min)** | **Alpha-cypermethrin at 225 nm** | **Deltamethrin at 233 nm** | **Lambda-cyhalothrin at 233 nm** | **Permethrin at 215 nm** |
| --- | --- | --- | --- | --- |
| 0-3 | 45% methanol:55% water | 45% acetonitrile:55% water | 45% acetonitrile:55% water | 90% water:10% methanol |
| 3-4.5 | 60% methanol:40% water | 60% acetonitrile:40% water | 60% acetonitrile:40% water | 80% water:20% methanol |
| 4.5-10 | 90% methanol:10% water | 92% acetonitrile:8% water | 90% acetonitrile:10% water | - |
| 4.5-5 | - | - | - | 70% water:30% methanol |
| 5-9 | - | - | - | 10% water:90% methanol |

**Table S3.** Experimental factorial design for preparation and optimization of deltamethrin nanoemulsions

| **Code** | **a.i of pyrethroid** | | **DMSO** | | **Tween 80** | | **Water** | | **Total volume (mL)** | **Sonication pulses (cycle/sec)** | **Sonication time (min)** | **Sonication power (%, Hz)** |
| --- | --- | --- | --- | --- | --- | --- | --- | --- | --- | --- | --- | --- |
|  | **%** | **g** | **%** | **mL** | **%** | **mL** | **%** | **mL** |  |  |  |  |
| **1** | 0.5 | 0.125 | 44 | 11.0 | 15.0 | 3.75 | 40.5 | 10.125 | 25 | 9 | 15 | 75 |
| **2** | 2.5 | 0.625 | 44 | 11.0 | 15.0 | 3.75 | 38.5 | 9.625 | 25 | 5 | 5 | 75 |
| **3** | 0.5 | 0.125 | 50 | 12.5 | 15.0 | 3.75 | 34.5 | 8.625 | 25 | 5 | 15 | 25 |
| **4** | 2.5 | 0.625 | 50 | 12.5 | 15.0 | 3.75 | 32.5 | 8.125 | 25 | 9 | 5 | 25 |
| **5** | 0.5 | 0.125 | 44 | 11.0 | 20.0 | 5.00 | 35.5 | 8.875 | 25 | 9 | 5 | 25 |
| **6** | 2.5 | 0.625 | 44 | 11.0 | 20.0 | 5.00 | 33.5 | 8.375 | 25 | 5 | 15 | 25 |
| **7** | 0.5 | 0.125 | 50 | 12.5 | 20.0 | 5.00 | 29.5 | 7.375 | 25 | 5 | 5 | 75 |
| **8** | 2.5 | 0.625 | 50 | 12.5 | 20.0 | 5.00 | 27.5 | 6.875 | 25 | 9 | 15 | 75 |
| **9** | 1.5 | 0.375 | 47 | 11.75 | 17.5 | 4.375 | 34.0 | 8.50 | 25 | 7 | 10 | 50 |

**Table S4.** The observed visual stability, droplet size, polydispersity index (PDI), zeta potential, dynamic (absolute) viscosity, and pH of prepared deltamethrin nanoemulsions

| **Code** | **Visual appearance** | **Droplet size**  **(nm) ±SE** | **Polydispersity index**  **(PDI) ±SE** | **Viscosity**  **(mPa.s)±SE** | **pH** | **Stability after** | |
| --- | --- | --- | --- | --- | --- | --- | --- |
|  |  |  |  |  |  | **Centrifugation**  **at 5000 rpm** | **Heating-cooling cycle** |
| **1** | Clear | 172^i^±34.07 | 0.827^a^**±**0.10 | 74.67^c^±7.86 | 7.84 | **√** | × |
| **2** | Milky | 8925^a^±2.60 | 0.158^b^**±**0.04 | 80.16^c^±0.60 | 7.89 | × | × |
| **3** | Clear | 1360^d^±5.81 | 0.897^a^**±**0.05 | 40.32^e^±5.77 | 8.18 | × | × |
| **4** | Clear | 3090^b^±18.35 | 0.944^a^**±**0.06 | 81.00^c^±0.33 | 8.15 | × | × |
| **5** | Clear | 364^h^±12.05 | 0.711^a^**±**0.12 | 90.23^c^±0.82 | 8.12 | **√** | × |
| **6** | Milky | 1102^e^±21.78 | 0.813^a^**±**0.19 | 90.23^c^±0.23 | 8.00 | × | × |
| **7** | Clear | 417^g^±5.46 | 0.516^a^**±**0.11 | 37.67^e^±3.33 | 8.15 | × | × |
| **8** | Clear | 1447^c^±9.32 | 0.767^a^**±**0.14 | 160.23^b^±5.77 | 8.10 | × | × |
| **9** | Milky | 1003^f^±3.33 | 0.939^a^**±**0.03 | 70.67^d^±1.12 | 7.78 | × | × |

Different letters in the same column indicate significant differences according to the Student-Newman-Keuls (SNK) test (*P* ≤ 0.05). (√) refer to the stable state, (×) refer to non-stable state ((powder participate (pp), colloids (coll) or oil and water layers (O/W)), color milky or transparent.

**
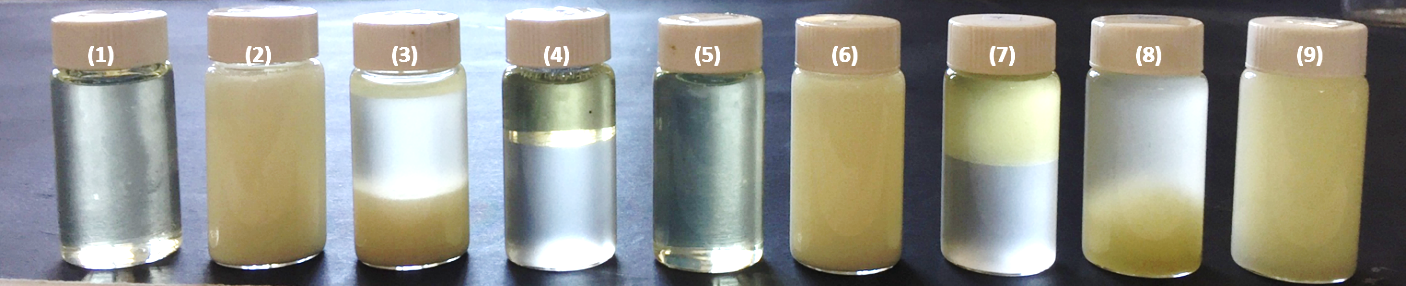
**

**Figure S1.** The visual appearance of prepared deltamethrin nanoemulsions. The code number represents the experimental factorial design shown in Table 2**.**

**Figure S2.** Zeta potential distribution graph of pyrethroid nanoemulsions of alpha-cypermethrin (A), deltamethrin (B), lambda-cyhalothrin (C), and permethrin (D).

**
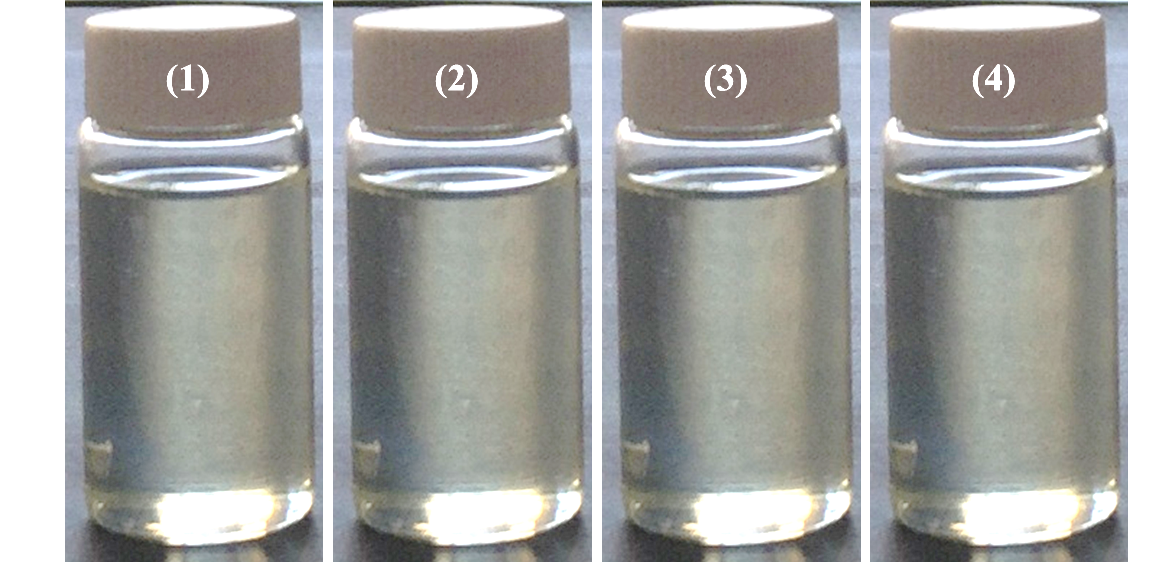
**

**Figure S3.** The visual appearance of pyrethroid nanoemulsions of alpha-cypermethrin (1), deltamethrin (2), lambda-cyhalothrin (3), and permethrin (4).

**Figure S4.** Docking view of the tested pyrethroids on the binding sites of ATPase (PDB ID: 4byg). Alpha-cypermethrin (A), lambda-cyhalothrin (B), deltamethrin, (C), and permethrin (D). Left is the 2D interaction diagram structure and right is the complex structure in stereo view (3D).

**(C)**


**Figure S5.** Docking view of the tested pyrethroids on the binding sites of CaE (PDB ID: 5w1u). Alpha-cypermethrin (A), lambda-cyhalothrin (B), deltamethrin, (C), and permethrin (D). Left is the 2D interaction diagram structure, and right is the complex structure in stereo view (3D).

**(C)**

**Figure S6.** Docking view of the tested pyrethroids on the binding sites of GST (PDB ID: 5ft3). Alpha-cypermethrin (A), lambda-cyhalothrin (B), deltamethrin, (C), and permethrin (D). Left is the 2D interaction diagram structure, and right is the complex structure in stereo view (3D).

**(C)**
